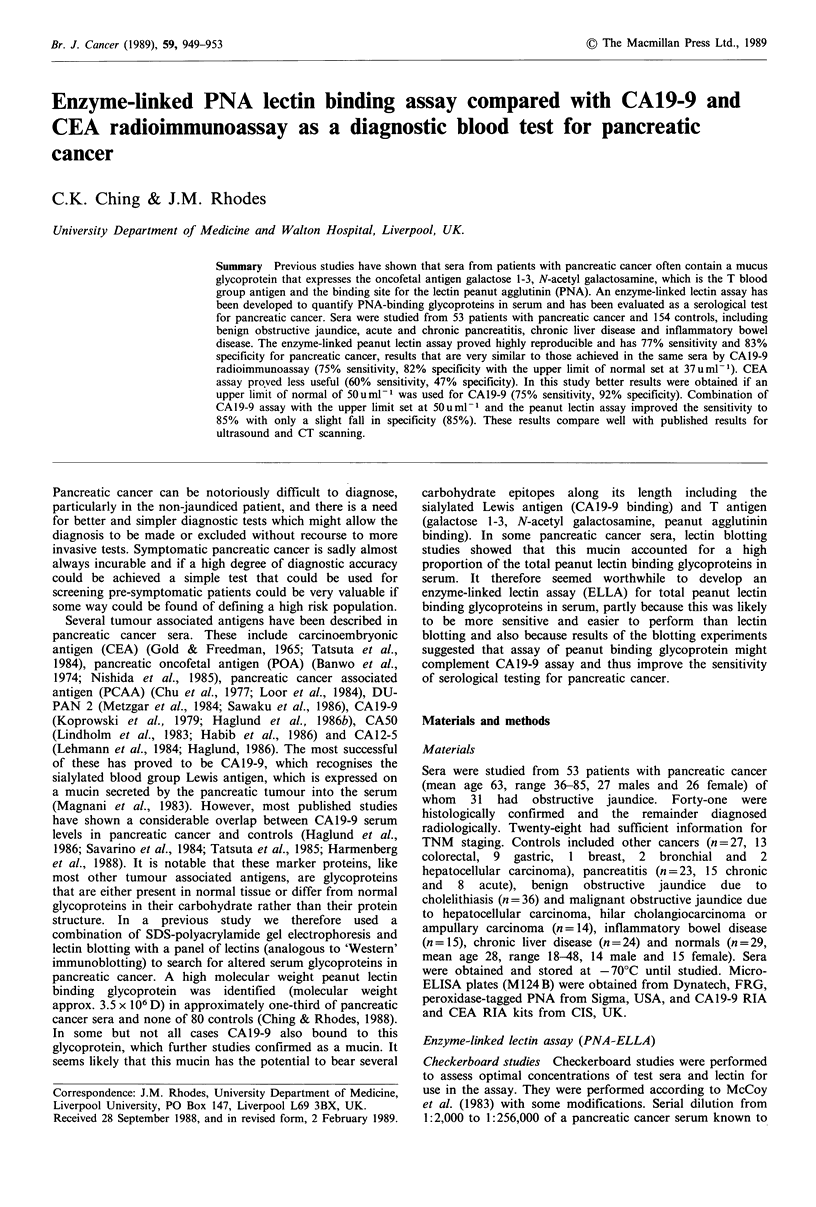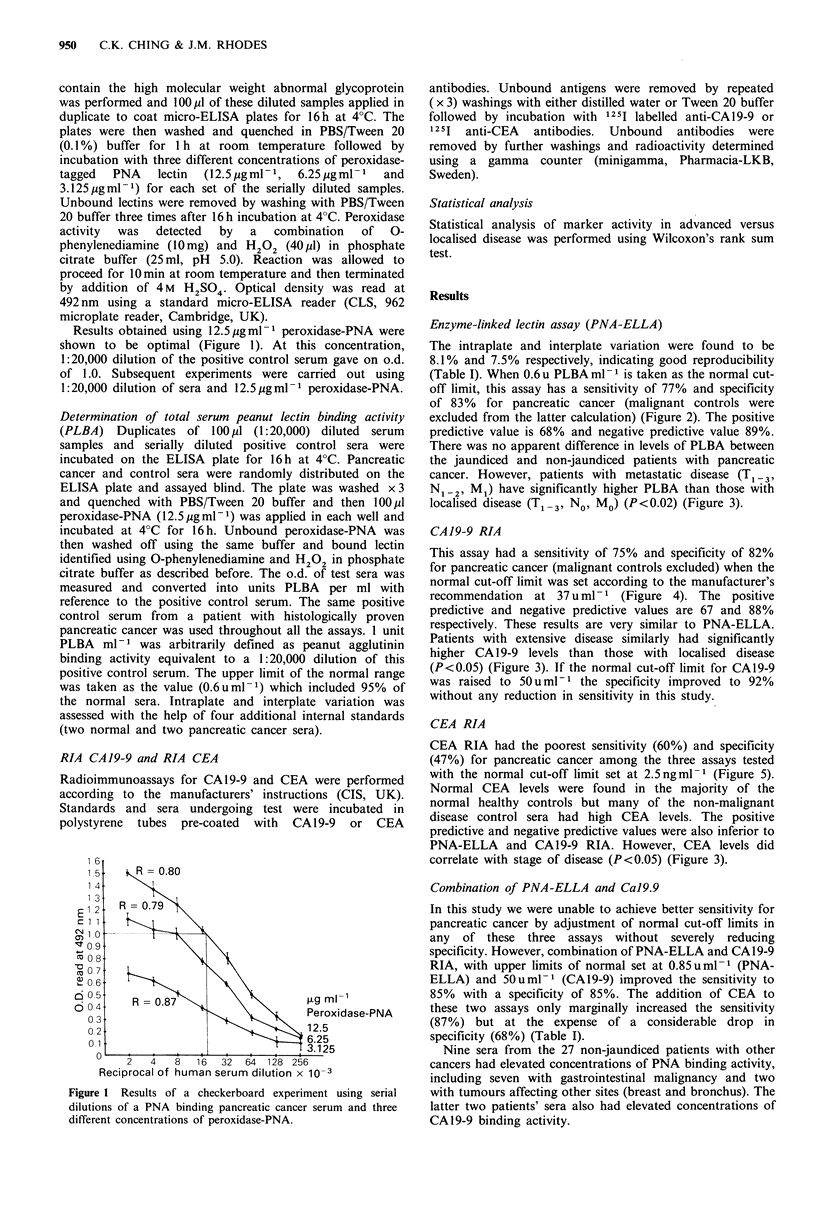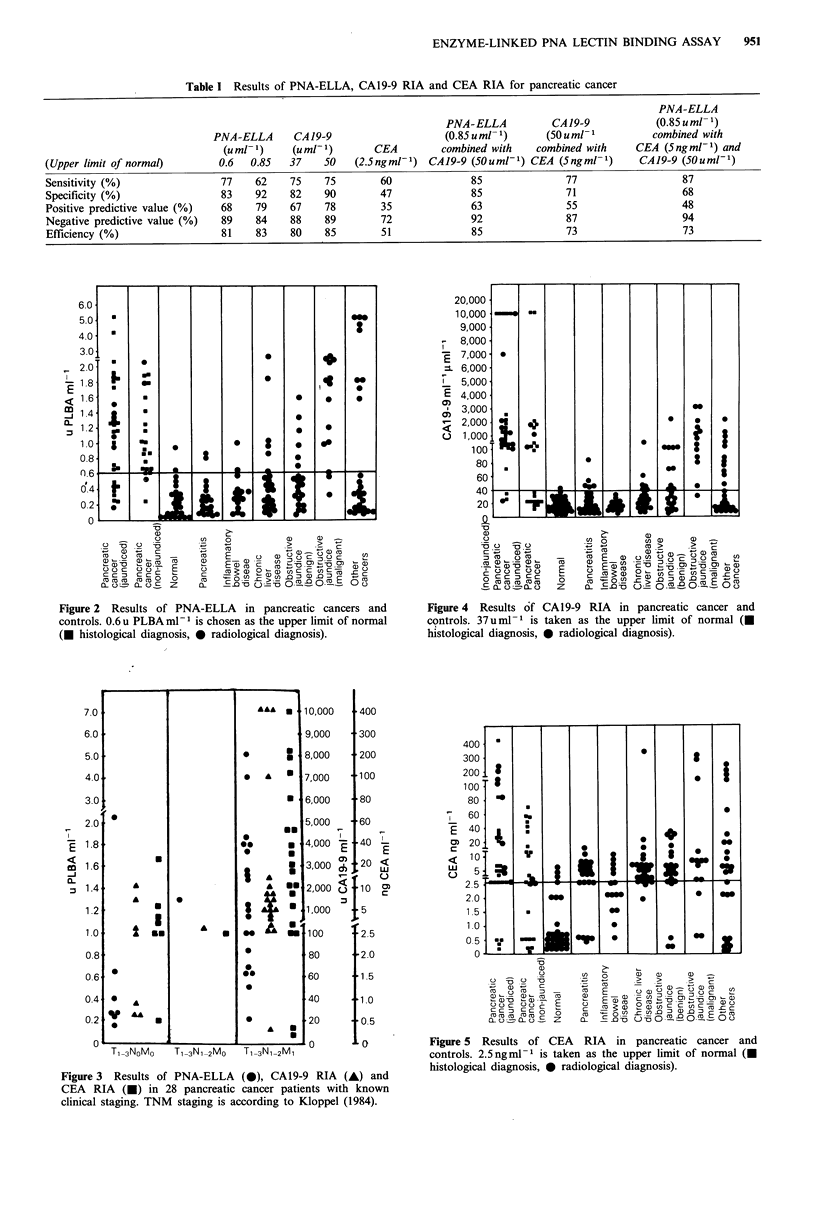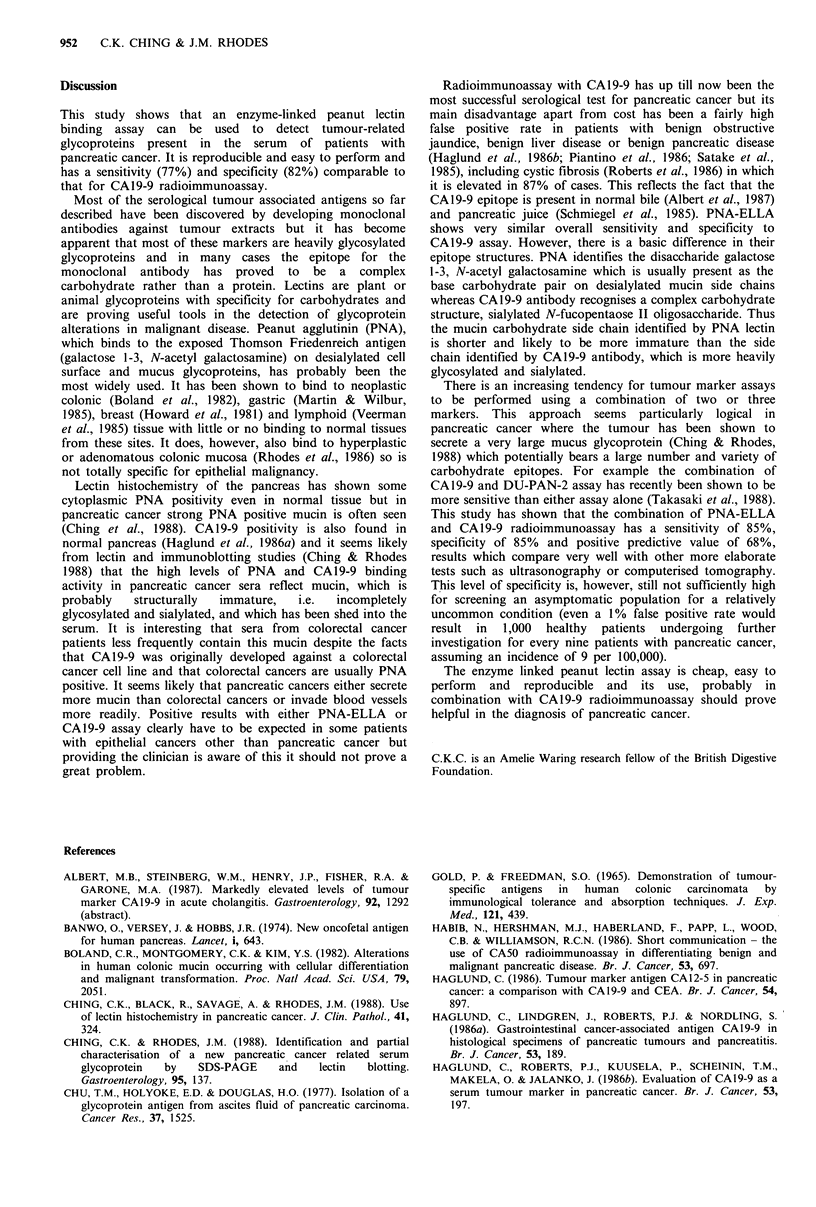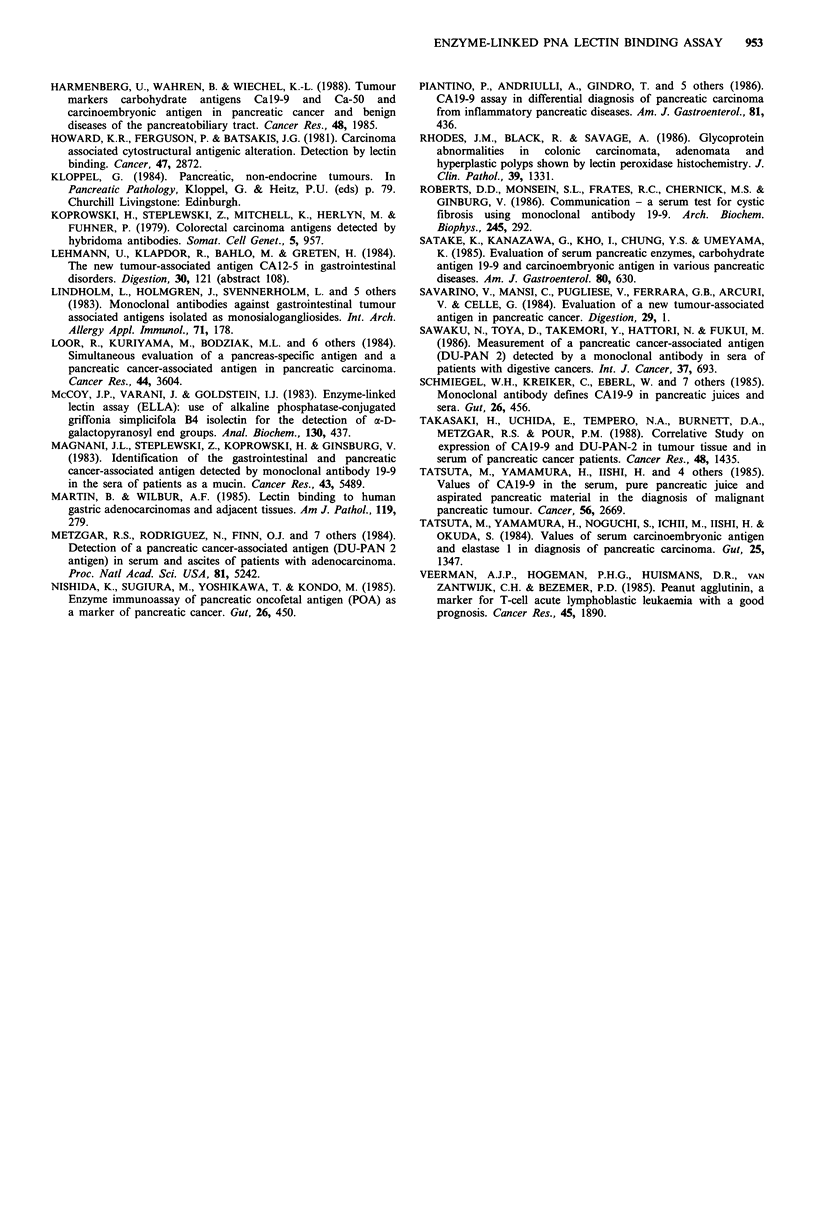# Enzyme-linked PNA lectin binding assay compared with CA19-9 and CEA radioimmunoassay as a diagnostic blood test for pancreatic cancer.

**DOI:** 10.1038/bjc.1989.202

**Published:** 1989-06

**Authors:** C. K. Ching, J. M. Rhodes

**Affiliations:** University Department of Medicine, Liverpool University, UK.

## Abstract

Previous studies have shown that sera from patients with pancreatic cancer often contain a mucus glycoprotein that expresses the oncofetal antigen galactose 1-3, N-acetyl galactosamine, which is the T blood group antigen and the binding site for the lectin peanut agglutinin (PNA). An enzyme-linked lectin assay has been developed to quantify PNA-binding glycoproteins in serum and has been evaluated as a serological test for pancreatic cancer. Sera were studied from 53 patients with pancreatic cancer and 154 controls, including benign obstructive jaundice, acute and chronic pancreatitis, chronic liver disease and inflammatory bowel disease. The enzyme-linked peanut lectin assay proved highly reproducible and has 77% sensitivity and 83% specificity for pancreatic cancer, results that are very similar to those achieved in the same sera by CA19-9 radioimmunoassay (75% sensitivity, 82% specificity with the upper limit of normal set at 37 u ml-1). CEA assay proved less useful (60% sensitivity, 47% specificity). In this study better results were obtained if an upper limit of normal of 50 u ml-1 was used for CA19-9 (75% sensitivity, 92% specificity). Combination of CA19-9 assay with the upper limit set at 50 u ml-1 and the peanut lectin assay improved the sensitivity to 85% with only a slight fall in specificity (85%). These results compare well with published results for ultrasound and CT scanning.


Br. J. Cancer (1989), 59, 949–953 © The Macmillan Press Ltd., 1989

### Enzyme-linked PNA lectin binding assay compared with CA19-9 and CEA radioimmunoassay as a diagnostic blood test for pancreatic cancer

C.K. Ching & J.M. Rhodes

*University Department of Medicine and Walton Hospital, Liverpool, UK.*

**Summary** Previous studies have shown that sera from patients with pancreatic cancer often contain a mucus glycoprotein that expresses the oncofetal antigen galactose 1-3, *N*-acetyl galactosamine, which is the T blood group antigen and the binding site for the lectin peanut agglutinin (PNA). An enzyme-linked lectin assay has been developed to quantify PNA-binding glycoproteins in serum and has been evaluated as a serological test for pancreatic cancer. Sera were studied from 53 patients with pancreatic cancer and 154 controls, including benign obstructive jaundice, acute and chronic pancreatitis, chronic liver disease and inflammatory bowel disease. The enzyme-linked peanut lectin assay proved highly reproducible and has 77% sensitivity and 83% specificity for pancreatic cancer, results that are very similar to those achieved in the same sera by CA19-9 radioimmunoassay (75% sensitivity, 82% specificity with the upper limit of normal set at $37\,u\,ml^{-1}$). CEA assay proved less useful (60% sensitivity, 47% specificity). In this study better results were obtained if an upper limit of normal of $50\,u\,ml^{-1}$ was used for CA19-9 (75% sensitivity, 92% specificity). Combination of CA19-9 assay with the upper limit set at $50\,u\,ml^{-1}$ and the peanut lectin assay improved the sensitivity to 85% with only a slight fall in specificity (85%). These results compare well with published results for ultrasound and CT scanning.

Pancreatic cancer can be notoriously difficult to diagnose, particularly in the non-jaundiced patient, and there is a need for better and simpler diagnostic tests which might allow the diagnosis to be made or excluded without recourse to more invasive tests. Symptomatic pancreatic cancer is sadly almost always incurable and if a high degree of diagnostic accuracy could be achieved a simple test that could be used for screening pre-symptomatic patients could be very valuable if some way could be found of defining a high risk population.

Several tumour associated antigens have been described in pancreatic cancer sera. These include carcinoembryonic antigen (CEA) (Gold & Freedman, 1965; Tatsuta *et al.*, 1984), pancreatic oncofetal antigen (POA) (Banwo *et al.*, 1974; Nishida *et al.*, 1985), pancreatic cancer associated antigen (PCAA) (Chu *et al.*, 1977; Loor *et al.*, 1984), DU-PAN 2 (Metzgar *et al.*, 1984; Sawaku *et al.*, 1986), CA19-9 (Koprowski *et al.*, 1979; Haglund *et al.*, 1986*b*), CA50 (Lindholm *et al.*, 1983; Habib *et al.*, 1986) and CA12-5 (Lehmann *et al.*, 1984; Haglund, 1986). The most successful of these has proved to be CA19-9, which recognises the sialylated blood group Lewis antigen, which is expressed on a mucin secreted by the pancreatic tumour into the serum (Magnani *et al.*, 1983). However, most published studies have shown a considerable overlap between CA19-9 serum levels in pancreatic cancer and controls (Haglund *et al.*, 1986; Savarino *et al.*, 1984; Tatsuta *et al.*, 1985; Harmenberg *et al.*, 1988). It is notable that these marker proteins, like most other tumour associated antigens, are glycoproteins that are either present in normal tissue or differ from normal glycoproteins in their carbohydrate rather than their protein structure. In a previous study we therefore used a combination of SDS-polyacrylamide gel electrophoresis and lectin blotting with a panel of lectins (analogous to 'Western' immunoblotting) to search for altered serum glycoproteins in pancreatic cancer. A high molecular weight peanut lectin binding glycoprotein was identified (molecular weight approx. $3.5 \times 10^6$ D) in approximately one-third of pancreatic cancer sera and none of 80 controls (Ching & Rhodes, 1988). In some but not all cases CA19-9 also bound to this glycoprotein, which further studies confirmed as a mucin. It seems likely that this mucin has the potential to bear several carbohydrate epitopes along its length including the sialylated Lewis antigen (CA19-9 binding) and T antigen (galactose 1-3, *N*-acetyl galactosamine, peanut agglutinin binding). In some pancreatic cancer sera, lectin blotting studies showed that this mucin accounted for a high proportion of the total peanut lectin binding glycoproteins in serum. It therefore seemed worthwhile to develop an enzyme-linked lectin assay (ELLA) for total peanut lectin binding glycoproteins in serum, partly because this was likely to be more sensitive and easier to perform than lectin blotting and also because results of the blotting experiments suggested that assay of peanut binding glycoprotein might complement CA19-9 assay and thus improve the sensitivity of serological testing for pancreatic cancer.

Correspondence: J.M. Rhodes, University Department of Medicine, Liverpool University, PO Box 147, Liverpool L69 3BX, UK.
Received 28 September 1988, and in revised form, 2 February 1989.

### Materials and methods

### *Materials*

Sera were studied from 53 patients with pancreatic cancer (mean age 63, range 36–85, 27 males and 26 female) of whom 31 had obstructive jaundice. Forty-one were histologically confirmed and the remainder diagnosed radiologically. Twenty-eight had sufficient information for TNM staging. Controls included other cancers ($n=27$, 13 colorectal, 9 gastric, 1 breast, 2 bronchial and 2 hepatocellular carcinoma), pancreatitis ($n=23$, 15 chronic and 8 acute), benign obstructive jaundice due to cholelithiasis ($n=36$) and malignant obstructive jaundice due to hepatocellular carcinoma, hilar cholangiocarcinoma or ampullary carcinoma ($n=14$), inflammatory bowel disease ($n=15$), chronic liver disease ($n=24$) and normals ($n=29$, mean age 28, range 18–48, 14 male and 15 female). Sera were obtained and stored at $-70°C$ until studied. Micro-ELISA plates (M124B) were obtained from Dynatech, FRG, peroxidase-tagged PNA from Sigma, USA, and CA19-9 RIA and CEA RIA kits from CIS, UK.

### *Enzyme-linked lectin assay (PNA-ELLA)*

*Checkerboard studies* Checkerboard studies were performed to assess optimal concentrations of test sera and lectin for use in the assay. They were performed according to McCoy *et al.* (1983) with some modifications. Serial dilution from 1:2,000 to 1:256,000 of a pancreatic cancer serum known to contain the high molecular weight abnormal glycoprotein was performed and 100 μl of these diluted samples applied in duplicate to coat micro-ELISA plates for 16 h at 4°C. The plates were then washed and quenched in PBS/Tween 20 (0.1%) buffer for 1 h at room temperature followed by incubation with three different concentrations of peroxidase-tagged PNA lectin (12.5 μg ml$^{-1}$, 6.25 μg ml$^{-1}$ and 3.125 μg ml$^{-1}$) for each set of the serially diluted samples. Unbound lectins were removed by washing with PBS/Tween 20 buffer three times after 16 h incubation at 4°C. Peroxidase activity was detected by a combination of O-phenylenediamine (10 mg) and $H_2O_2$ (40 μl) in phosphate citrate buffer (25 ml, pH 5.0). Reaction was allowed to proceed for 10 min at room temperature and then terminated by addition of 4 M $H_2SO_4$. Optical density was read at 492 nm using a standard micro-ELISA reader (CLS, 962 microplate reader, Cambridge, UK).

Results obtained using 12.5 μg ml$^{-1}$ peroxidase-PNA were shown to be optimal (Figure 1). At this concentration, 1:20,000 dilution of the positive control serum gave on o.d. of 1.0. Subsequent experiments were carried out using 1:20,000 dilution of sera and 12.5 μg ml$^{-1}$ peroxidase-PNA.

*Determination of total serum peanut lectin binding activity (PLBA)* Duplicates of 100 μl (1:20,000) diluted serum samples and serially diluted positive control sera were incubated on the ELISA plate for 16 h at 4°C. Pancreatic cancer and control sera were randomly distributed on the ELISA plate and assayed blind. The plate was washed ×3 and quenched with PBS/Tween 20 buffer and then 100 μl peroxidase-PNA (12.5 μg ml$^{-1}$) was applied in each well and incubated at 4°C for 16 h. Unbound peroxidase-PNA was then washed off using the same buffer and bound lectin identified using O-phenylenediamine and $H_2O_2$ in phosphate citrate buffer as described before. The o.d. of test sera was measured and converted into units PLBA per ml with reference to the positive control serum. The same positive control serum from a patient with histologically proven pancreatic cancer was used throughout all the assays. 1 unit PLBA ml$^{-1}$ was arbitrarily defined as peanut agglutinin binding activity equivalent to a 1:20,000 dilution of this positive control serum. The upper limit of the normal range was taken as the value (0.6 u ml$^{-1}$) which included 95% of the normal sera. Intraplate and interplate variation was assessed with the help of four additional internal standards (two normal and two pancreatic cancer sera).

*RIA CA19-9 and RIA CEA*

Radioimmunoassays for CA19-9 and CEA were performed according to the manufacturers' instructions (CIS, UK). Standards and sera undergoing test were incubated in polystyrene tubes pre-coated with CA19-9 or CEA

**Figure 1** Results of a checkerboard experiment using serial dilutions of a PNA binding pancreatic cancer serum and three different concentrations of peroxidase-PNA.

antibodies. Unbound antigens were removed by repeated (×3) washings with either distilled water or Tween 20 buffer followed by incubation with $^{125}I$ labelled anti-CA19-9 or $^{125}I$ anti-CEA antibodies. Unbound antibodies were removed by further washings and radioactivity determined using a gamma counter (minigamma, Pharmacia-LKB, Sweden).

*Statistical analysis*

Statistical analysis of marker activity in advanced versus localised disease was performed using Wilcoxon's rank sum test.

### Results

*Enzyme-linked lectin assay (PNA-ELLA)*

The intraplate and interplate variation were found to be 8.1% and 7.5% respectively, indicating good reproducibility (Table I). When 0.6 u PLBA ml$^{-1}$ is taken as the normal cut-off limit, this assay has a sensitivity of 77% and specificity of 83% for pancreatic cancer (malignant controls were excluded from the latter calculation) (Figure 2). The positive predictive value is 68% and negative predictive value 89%. There was no apparent difference in levels of PLBA between the jaundiced and non-jaundiced patients with pancreatic cancer. However, patients with metastatic disease ($T_{1-3}$, $N_{1-2}$, $M_1$) have significantly higher PLBA than those with localised disease ($T_{1-3}$, $N_0$, $M_0$) ($P<0.02$) (Figure 3).

*CA19-9 RIA*

This assay had a sensitivity of 75% and specificity of 82% for pancreatic cancer (malignant controls excluded) when the normal cut-off limit was set according to the manufacturer's recommendation at 37 u ml$^{-1}$ (Figure 4). The positive predictive and negative predictive values are 67 and 88% respectively. These results are very similar to PNA-ELLA. Patients with extensive disease similarly had significantly higher CA19-9 levels than those with localised disease ($P<0.05$) (Figure 3). If the normal cut-off limit for CA19-9 was raised to 50 u ml$^{-1}$ the specificity improved to 92% without any reduction in sensitivity in this study.

*CEA RIA*

CEA RIA had the poorest sensitivity (60%) and specificity (47%) for pancreatic cancer among the three assays tested with the normal cut-off limit set at 2.5 ng ml$^{-1}$ (Figure 5). Normal CEA levels were found in the majority of the normal healthy controls but many of the non-malignant disease control sera had high CEA levels. The positive predictive and negative predictive values were also inferior to PNA-ELLA and CA19-9 RIA. However, CEA levels did correlate with stage of disease ($P<0.05$) (Figure 3).

*Combination of PNA-ELLA and Ca19.9*

In this study we were unable to achieve better sensitivity for pancreatic cancer by adjustment of normal cut-off limits in any of these three assays without severely reducing specificity. However, combination of PNA-ELLA and CA19-9 RIA, with upper limits of normal set at 0.85 u ml$^{-1}$ (PNA-ELLA) and 50 u ml$^{-1}$ (CA19-9) improved the sensitivity to 85% with a specificity of 85%. The addition of CEA to these two assays only marginally increased the sensitivity (87%) but at the expense of a considerable drop in specificity (68%) (Table I).

Nine sera from the 27 non-jaundiced patients with other cancers had elevated concentrations of PNA binding activity, including seven with gastrointestinal malignancy and two with tumours affecting other sites (breast and bronchus). The latter two patients' sera also had elevated concentrations of CA19-9 binding activity.

**Table I** Results of PNA-ELLA, CA19-9 RIA and CEA RIA for pancreatic cancer

| *(Upper limit of normal)* | *PNA-ELLA* ($u\,ml^{-1}$) *0.6* | *PNA-ELLA* ($u\,ml^{-1}$) *0.85* | *CA19-9* ($u\,ml^{-1}$) *37* | *CA19-9* ($u\,ml^{-1}$) *50* | *CEA* ($2.5\,ng\,ml^{-1}$) | *PNA-ELLA* ($0.85\,u\,ml^{-1}$) *combined with CA19-9* ($50\,u\,ml^{-1}$) | *CA19-9* ($50\,u\,ml^{-1}$) *combined with CEA* ($5\,ng\,ml^{-1}$) | *PNA-ELLA* ($0.85\,u\,ml^{-1}$) *combined with CEA* ($5\,ng\,ml^{-1}$) *and CA19-9* ($50\,u\,ml^{-1}$) |
|---|---|---|---|---|---|---|---|---|
| Sensitivity (%) | 77 | 62 | 75 | 75 | 60 | 85 | 77 | 87 |
| Specificity (%) | 83 | 92 | 82 | 90 | 47 | 85 | 71 | 68 |
| Positive predictive value (%) | 68 | 79 | 67 | 78 | 35 | 63 | 55 | 48 |
| Negative predictive value (%) | 89 | 84 | 88 | 89 | 72 | 92 | 87 | 94 |
| Efficiency (%) | 81 | 83 | 80 | 85 | 51 | 85 | 73 | 73 |

**Figure 2** Results of PNA-ELLA in pancreatic cancers and controls. 0.6 u PLBA $ml^{-1}$ is chosen as the upper limit of normal (■ histological diagnosis, ● radiological diagnosis).

**Figure 4** Results of CA19-9 RIA in pancreatic cancer and controls. 37 u $ml^{-1}$ is taken as the upper limit of normal (■ histological diagnosis, ● radiological diagnosis).

**Figure 3** Results of PNA-ELLA (●), CA19-9 RIA (▲) and CEA RIA (■) in 28 pancreatic cancer patients with known clinical staging. TNM staging is according to Kloppel (1984).

**Figure 5** Results of CEA RIA in pancreatic cancer and controls. 2.5 ng $ml^{-1}$ is taken as the upper limit of normal (■ histological diagnosis, ● radiological diagnosis).

### Discussion

This study shows that an enzyme-linked peanut lectin binding assay can be used to detect tumour-related glycoproteins present in the serum of patients with pancreatic cancer. It is reproducible and easy to perform and has a sensitivity (77%) and specificity (82%) comparable to that for CA19-9 radioimmunoassay.

Most of the serological tumour associated antigens so far described have been discovered by developing monoclonal antibodies against tumour extracts but it has become apparent that most of these markers are heavily glycosylated glycoproteins and in many cases the epitope for the monoclonal antibody has proved to be a complex carbohydrate rather than a protein. Lectins are plant or animal glycoproteins with specificity for carbohydrates and are proving useful tools in the detection of glycoprotein alterations in malignant disease. Peanut agglutinin (PNA), which binds to the exposed Thomson Friedenreich antigen (galactose 1-3, *N*-acetyl galactosamine) on desialylated cell surface and mucus glycoproteins, has probably been the most widely used. It has been shown to bind to neoplastic colonic (Boland *et al.*, 1982), gastric (Martin & Wilbur, 1985), breast (Howard *et al.*, 1981) and lymphoid (Veerman *et al.*, 1985) tissue with little or no binding to normal tissues from these sites. It does, however, also bind to hyperplastic or adenomatous colonic mucosa (Rhodes *et al.*, 1986) so is not totally specific for epithelial malignancy.

Lectin histochemistry of the pancreas has shown some cytoplasmic PNA positivity even in normal tissue but in pancreatic cancer strong PNA positive mucin is often seen (Ching *et al.*, 1988). CA19-9 positivity is also found in normal pancreas (Haglund *et al.*, 1986*a*) and it seems likely from lectin and immunoblotting studies (Ching & Rhodes 1988) that the high levels of PNA and CA19-9 binding activity in pancreatic cancer sera reflect mucin, which is probably structurally immature, i.e. incompletely glycosylated and sialylated, and which has been shed into the serum. It is interesting that sera from colorectal cancer patients less frequently contain this mucin despite the facts that CA19-9 was originally developed against a colorectal cancer cell line and that colorectal cancers are usually PNA positive. It seems likely that pancreatic cancers either secrete more mucin than colorectal cancers or invade blood vessels more readily. Positive results with either PNA-ELLA or CA19-9 assay clearly have to be expected in some patients with epithelial cancers other than pancreatic cancer but providing the clinician is aware of this it should not prove a great problem.

Radioimmunoassay with CA19-9 has up till now been the most successful serological test for pancreatic cancer but its main disadvantage apart from cost has been a fairly high false positive rate in patients with benign obstructive jaundice, benign liver disease or benign pancreatic disease (Haglund *et al.*, 1986*b*; Piantino *et al.*, 1986; Satake *et al.*, 1985), including cystic fibrosis (Roberts *et al.*, 1986) in which it is elevated in 87% of cases. This reflects the fact that the CA19-9 epitope is present in normal bile (Albert *et al.*, 1987) and pancreatic juice (Schmiegel *et al.*, 1985). PNA-ELLA shows very similar overall sensitivity and specificity to CA19-9 assay. However, there is a basic difference in their epitope structures. PNA identifies the disaccharide galactose 1-3, *N*-acetyl galactosamine which is usually present as the base carbohydrate pair on desialylated mucin side chains whereas CA19-9 antibody recognises a complex carbohydrate structure, sialylated *N*-fucopentaose II oligosaccharide. Thus the mucin carbohydrate side chain identified by PNA lectin is shorter and likely to be more immature than the side chain identified by CA19-9 antibody, which is more heavily glycosylated and sialylated.

There is an increasing tendency for tumour marker assays to be performed using a combination of two or three markers. This approach seems particularly logical in pancreatic cancer where the tumour has been shown to secrete a very large mucus glycoprotein (Ching & Rhodes, 1988) which potentially bears a large number and variety of carbohydrate epitopes. For example the combination of CA19-9 and DU-PAN-2 assay has recently been shown to be more sensitive than either assay alone (Takasaki *et al.*, 1988). This study has shown that the combination of PNA-ELLA and CA19-9 radioimmunoassay has a sensitivity of 85%, specificity of 85% and positive predictive value of 68%, results which compare very well with other more elaborate tests such as ultrasonography or computerised tomography. This level of specificity is, however, still not sufficiently high for screening an asymptomatic population for a relatively uncommon condition (even a 1% false positive rate would result in 1,000 healthy patients undergoing further investigation for every nine patients with pancreatic cancer, assuming an incidence of 9 per 100,000).

The enzyme linked peanut lectin assay is cheap, easy to perform and reproducible and its use, probably in combination with CA19-9 radioimmunoassay should prove helpful in the diagnosis of pancreatic cancer.

C.K.C. is an Amelie Waring research fellow of the British Digestive Foundation.

### References

ALBERT, M.B., STEINBERG, W.M., HENRY, J.P., FISHER, R.A. & GARONE, M.A. (1987). Markedly elevated levels of tumour marker CA19-9 in acute cholangitis. *Gastroenterology*, **92**, 1292 (abstract).

BANWO, O., VERSEY, J. & HOBBS, J.R. (1974). New oncofetal antigen for human pancreas. *Lancet*, **i**, 643.

BOLAND, C.R., MONTGOMERY, C.K. & KIM, Y.S. (1982). Alterations in human colonic mucin occurring with cellular differentiation and malignant transformation. *Proc. Natl Acad. Sci. USA*, **79**, 2051.

CHING, C.K., BLACK, R., SAVAGE, A. & RHODES, J.M. (1988). Use of lectin histochemistry in pancreatic cancer. *J. Clin. Pathol.*, **41**, 324.

CHING, C.K. & RHODES, J.M. (1988). Identification and partial characterisation of a new pancreatic cancer related serum glycoprotein by SDS-PAGE and lectin blotting. *Gastroenterology*, **95**, 137.

CHU, T.M., HOLYOKE, E.D. & DOUGLAS, H.O. (1977). Isolation of a glycoprotein antigen from ascites fluid of pancreatic carcinoma. *Cancer Res.*, **37**, 1525.

GOLD, P. & FREEDMAN, S.O. (1965). Demonstration of tumour-specific antigens in human colonic carcinomata by immunological tolerance and absorption techniques. *J. Exp. Med.*, **121**, 439.

HABIB, N., HERSHMAN, M.J., HABERLAND, F., PAPP, L., WOOD, C.B. & WILLIAMSON, R.C.N. (1986). Short communication – the use of CA50 radioimmunoassay in differentiating benign and malignant pancreatic disease. *Br. J. Cancer*, **53**, 697.

HAGLUND, C. (1986). Tumour marker antigen CA12-5 in pancreatic cancer: a comparison with CA19-9 and CEA. *Br. J. Cancer*, **54**, 897.

HAGLUND, C., LINDGREN, J., ROBERTS, P.J. & NORDLING, S. (1986*a*). Gastrointestinal cancer-associated antigen CA19-9 in histological specimens of pancreatic tumours and pancreatitis. *Br. J. Cancer*, **53**, 189.

HAGLUND, C., ROBERTS, P.J., KUUSELA, P., SCHEININ, T.M., MAKELA, O. & JALANKO, J. (1986*b*). Evaluation of CA19-9 as a serum tumour marker in pancreatic cancer. *Br. J. Cancer*, **53**, 197.

HARMENBERG, U., WAHREN, B. & WIECHEL, K.-L. (1988). Tumour markers carbohydrate antigens Ca19-9 and Ca-50 and carcinoembryonic antigen in pancreatic cancer and benign diseases of the pancreatobiliary tract. *Cancer Res.*, **48**, 1985.

HOWARD, K.R., FERGUSON, P. & BATSAKIS, J.G. (1981). Carcinoma associated cytostructural antigenic alteration. Detection by lectin binding. *Cancer*, **47**, 2872.

KLOPPEL, G. (1984). Pancreatic, non-endocrine tumours. In *Pancreatic Pathology*, Kloppel, G. & Heitz, P.U. (eds) p. 79. Churchill Livingstone: Edinburgh.

KOPROWSKI, H., STEPLEWSKI, Z., MITCHELL, K., HERLYN, M. & FUHNER, P. (1979). Colorectal carcinoma antigens detected by hybridoma antibodies. *Somat. Cell Genet.*, **5**, 957.

LEHMANN, U., KLAPDOR, R., BAHLO, M. & GRETEN, H. (1984). The new tumour-associated antigen CA12-5 in gastrointestinal disorders. *Digestion*, **30**, 121 (abstract 108).

LINDHOLM, L., HOLMGREN, J., SVENNERHOLM, L. and 5 others (1983). Monoclonal antibodies against gastrointestinal tumour associated antigens isolated as monosialogangliosides. *Int. Arch. Allergy Appl. Immunol.*, **71**, 178.

LOOR, R., KURIYAMA, M., BODZIAK, M.L. and 6 others (1984). Simultaneous evaluation of a pancreas-specific antigen and a pancreatic cancer-associated antigen in pancreatic carcinoma. *Cancer Res.*, **44**, 3604.

McCOY, J.P., VARANI, J. & GOLDSTEIN, I.J. (1983). Enzyme-linked lectin assay (ELLA): use of alkaline phosphatase-conjugated griffonia simplicifola B4 isolectin for the detection of α-D-galactopyranosyl end groups. *Anal. Biochem.*, **130**, 437.

MAGNANI, J.L., STEPLEWSKI, Z., KOPROWSKI, H. & GINSBURG, V. (1983). Identification of the gastrointestinal and pancreatic cancer-associated antigen detected by monoclonal antibody 19-9 in the sera of patients as a mucin. *Cancer Res.*, **43**, 5489.

MARTIN, B. & WILBUR, A.F. (1985). Lectin binding to human gastric adenocarcinomas and adjacent tissues. *Am J. Pathol.*, **119**, 279.

METZGAR, R.S., RODRIGUEZ, N., FINN, O.J. and 7 others (1984). Detection of a pancreatic cancer-associated antigen (DU-PAN 2 antigen) in serum and ascites of patients with adenocarcinoma. *Proc. Natl Acad. Sci. USA*, **81**, 5242.

NISHIDA, K., SUGIURA, M., YOSHIKAWA, T. & KONDO, M. (1985). Enzyme immunoassay of pancreatic oncofetal antigen (POA) as a marker of pancreatic cancer. *Gut*, **26**, 450.

PIANTINO, P., ANDRIULLI, A., GINDRO, T. and 5 others (1986). CA19-9 assay in differential diagnosis of pancreatic carcinoma from inflammatory pancreatic diseases. *Am. J. Gastroenterol.*, **81**, 436.

RHODES, J.M., BLACK, R. & SAVAGE, A. (1986). Glycoprotein abnormalities in colonic carcinomata, adenomata and hyperplastic polyps shown by lectin peroxidase histochemistry. *J. Clin. Pathol.*, **39**, 1331.

ROBERTS, D.D., MONSEIN, S.L., FRATES, R.C., CHERNICK, M.S. & GINBURG, V. (1986). Communication – a serum test for cystic fibrosis using monoclonal antibody 19-9. *Arch. Biochem. Biophys.*, **245**, 292.

SATAKE, K., KANAZAWA, G., KHO, I., CHUNG, Y.S. & UMEYAMA, K. (1985). Evaluation of serum pancreatic enzymes, carbohydrate antigen 19-9 and carcinoembryonic antigen in various pancreatic diseases. *Am. J. Gastroenterol.* **80**, 630.

SAVARINO, V., MANSI, C., PUGLIESE, V., FERRARA, G.B., ARCURI, V. & CELLE, G. (1984). Evaluation of a new tumour-associated antigen in pancreatic cancer. *Digestion*, **29**, 1.

SAWAKU, N., TOYA, D., TAKEMORI, Y., HATTORI, N. & FUKUI, M. (1986). Measurement of a pancreatic cancer-associated antigen (DU-PAN 2) detected by a monoclonal antibody in sera of patients with digestive cancers. *Int. J. Cancer*, **37**, 693.

SCHMIEGEL, W.H., KREIKER, C., EBERL, W. and 7 others (1985). Monoclonal antibody defines CA19-9 in pancreatic juices and sera. *Gut*, **26**, 456.

TAKASAKI, H., UCHIDA, E., TEMPERO, N.A., BURNETT, D.A., METZGAR, R.S. & POUR, P.M. (1988). Correlative Study on expression of CA19-9 and DU-PAN-2 in tumour tissue and in serum of pancreatic cancer patients. *Cancer Res.*, **48**, 1435.

TATSUTA, M., YAMAMURA, H., IISHI, H. and 4 others (1985). Values of CA19-9 in the serum, pure pancreatic juice and aspirated pancreatic material in the diagnosis of malignant pancreatic tumour. *Cancer*, **56**, 2669.

TATSUTA, M., YAMAMURA, H., NOGUCHI, S., ICHII, M., IISHI, H. & OKUDA, S. (1984). Values of serum carcinoembryonic antigen and elastase 1 in diagnosis of pancreatic carcinoma. *Gut*, **25**, 1347.

VEERMAN, A.J.P., HOGEMAN, P.H.G., HUISMANS, D.R., VAN ZANTWIJK, C.H. & BEZEMER, P.D. (1985). Peanut agglutinin, a marker for T-cell acute lymphoblastic leukaemia with a good prognosis. *Cancer Res.*, **45**, 1890.